# Recent Advances in Photochargeable Integrated and All-in-One Supercapacitor Devices

**DOI:** 10.1021/acsomega.3c07464

**Published:** 2023-12-11

**Authors:** Cigdem Tuc Altaf, Arpad Mihai Rostas, Adriana Popa, Dana Toloman, Maria Stefan, Nurdan Demirci Sankir, Mehmet Sankir

**Affiliations:** † Department of Materials Science and Nanotechnology Engineering, TOBB University of Economics and Technology, Sogutozu Caddesi No 43 Sogutozu 06560 Ankara, Turkey; ‡ National Institute for Research and Development of Isotopic and Molecular Technologies- INCDTIM, 67-103 Donat, 400293 Cluj-Napoca, Romania

## Abstract

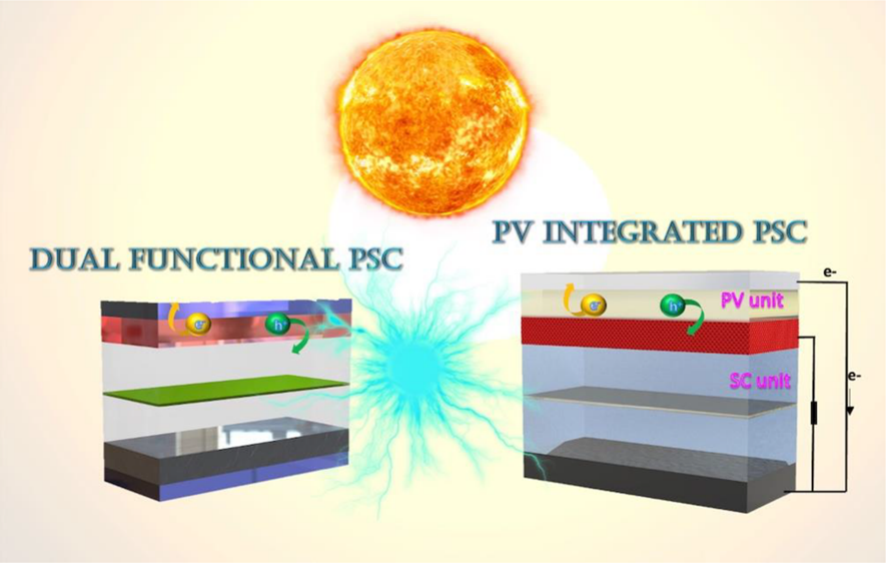

Photoassisted energy storage systems, which enable both the conversion and storage of solar energy, have attracted attention in recent years. These systems, which started about 20 years ago with the individual production of dye-sensitized solar cells and capacitors and their integration, today allow more compact and cost-effective designs using dual-acting electrodes. Solar-assisted batterylike or hybrid supercapacitors have also shown promise with their high energy densities. This review summarizes all of these device designs and conveys the cutting-edge studies in this field. Besides, this review aims to emphasize the effects of point, extrinsic, intrinsic, and 2D-planar defects on the performance of photoassisted energy storage systems since it is known that defect structures, as well as electrical, optical, and surface properties, affect the device performance. Here, it is also targeted to draw attention to how critical the design, material selection, and material properties are for these new-generation energy conversion and storage devices, which have a high potential to see commercial examples quickly and to be recognized by more readers.

## 1 Introduction

Research in the conversion and storage of renewable energy has gained vast acceleration since 2008 with the global energy crisis regarding environmental issues and economic depression due to the exploitation of carbon-based fuels.^1−3^ On the basis of the report of the International Energy Agency (IEA), the capacity of renewable energy is expected to double in the next five years to decrease the use of coal in the electricity generation process to keep global warming at 1.5 °C.^4^ Thanks to the enormous development in materials science and high-tech energy systems, many sustainable energy applications have been reported by incorporating hydropower, biomass, geothermal, wind, solar, and ocean energies.^5,6^ Among all, solar power is the most valuable renewable energy source as it is a continuous power supply to the Earth (3.8 × 10^23^ kW).^7,8^ Therefore, the scientific community has been urged to seek new developments to utilize solar power for energy conversion and storage applications, such as photovoltaic (PV) devices, typically solar cells;^9−14^ photoelectrochemical (PEC) H_2_ generation;^15−24^ photocatalytic systems;^25−30^ photorechargeable batteries;^31−34^ and optoelectronics.^35,36^ Although solar light can be used directly, its unbalanced intermittency regularly affects energy harvest, and a huge energy potential remains untapped. Thus, capturing, converting, and storing solar energy in a single and compact system is of great interest for long-term solution. In this context, emergent multifunctional devices, so-called photosupercapacitors (PSCs), simultaneously provide compact systems combining PV and supercapacitors (SCs) for photoelectric conversion and energy storage.^37−40^ To date, many types of PSCs that can be categorized into two main groups, integrated and all-in-one type PSC devices, have been reported. The predominantly studied PSCs are integrated PSCs composed of a solar energy converter and a storage part. Therefore, revising the current photovoltaic (PV) system progress is essential.

Silicon-based PVs are the first generation of PV devices and employ crystalline silicon (c-Si). Second-generation PVs involving a high cost with limited resources, such as GaAs, CdTe, and CIGS materials, are unsuitable for integrated PSC devices. Thus, at present, third/next-generation PVs, such as quantum dot (QD)-sensitized solar cells,^41,42^ dye-sensitized solar cells (DSCC),^43^ polymer solar cells,^44^ perovskites, and tandem solar cells,^45−47^ have been proven to be auspicious candidates for the design of PSC devices in a vast diversity of designs, including portable and wearable energy storage materials. The PV part of the PSC device demands a rigid or flexible transparent conducting substrate that is used as a platform for photoactive deposits. Depending on the design, the counter electrode (CE) of the SC and PV parts can be shared in the PSC device. The PV part is responsible for the photocharging process carried by light absorption. Therefore, the photoconversion efficiency is an important parameter for PV-integrated PSC devices. As the first candidate of third-generation PVs in the solar cell research area, DSSCs are a widely studied type because of their ease of manufacture and low cost. Although the maximum theoretical efficiency has been reported as 32%, the real-time efficiency of DSSCs has reached up to 15.2% in a recent study.^48^ Conversely, in QDSCs, nanocrystalline semiconductor quantum dots are utilized as photoactive material or sensitizers.^49^ Quantum dots combine the advantages of having a tunable bandgap depending on size and compositions and ease of production at low-cost, low-temperature solution-based processes.^50^ Undoubtedly, the most attractive PV materials of the recent years are the perovskites in both the academic and industrial field because of their remarkable photoconversion efficiencies reaching above 25%.^51^ OPV (or polymer PVs) have the active layer of carbon-based semiconductors, such as conjugated polymers. These materials are preferred because of their abundance, low-cost, nontoxicity, and ease of manufacturing on large areas and flexible substrates. The photoconversion efficiency of a single OPV module has reached up to ∼19% as a consequence of a serious consideration in academic research.

All-in-one PSCs are compact, single-unit systems that do not require an additional circuit for solar energy storage. On the contrary, solar energy can be captured, converted, and stored by photoactive materials and redox pairs.^52−55^ PSCs offer ease of assembly, low cost, high operating voltages, and extended cycling stability. Herein, this review aims to provide a brief analysis of the development of PSC devices by focusing on the different types of PSC devices constructed by solar cell integrated and all-in-one systems, their working principles and mechanisms, photoactive electrode materials, and improvements in their efficiencies by defect engineering.

## 2 Integrated Systems

A literature review on the recently developed integrated systems based on PV-integrated PSCs is provided in this section (Table 1). The current integrated PSC systems have been analyzed by dividing them into categories on the basis of PV component types. The efficiency parameters of an integrated device can be revealed by the PV energy conversion and electrochemical energy storage units using the overall energy conversion and storage efficiency (η_overall_) equation.^56^ The η_overall_ is the ratio between the energy stored during photo charge (*E*_storage_) and the energy produced during illumination (*E*_light_).^57^
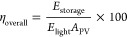
1
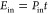
2where *A*_PV_ is the active area of the solar cell, and *E*_in_ and *P*_in_ are the amount of energy and power obtained during charging, respectively. For the η_overall_ parameter, first, the photoconversion efficiency (PCE, η_PSC_) of the solar cells unit under light illumination is evaluated using eqs 3 and 4)^58,59^
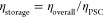
3
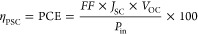
4where *FF*, *J*_SC_, *V*_OC_, and *P*_in_ represent the fill factor, the short-circuit current density, the open-circuit voltage, and the power density of the incident light, respectively.

**Table 1:** Literature Comparisons of the PV-Integrated PSC Devices

PV unit	SC unit	electrolyte	PCE (%)	capacitance	energy density	*t*_dis_ (s)
DSSC^64^	CNTs/MnO_2_	TEABF_4_/ACN	0.048	13.10 mF cm^–2^	-	∼380
DSSC^100^	MoS_2_	H_3_PO_4_/PVA	9.5	18.50 mF cm^–2^	1.65 μWh cm^–2^	∼25
DSSC^101^	rGO	BMIMTFSI/THF	2.8	0.14 mF cm^–2^	-	20
DSSC^102^	AC	Pyr_14_TFSI/PEO/BPN	2.25	0.017 mAh cm^–2^	-	1000
DSSC^63^	PPy/RGO	KOH/PVA	2.4	124.70 F g^–1^	26.6 Wh kg^–1^	20
DSSC^103^	PEDOT/CNTs	Nafion 117/H_2_SO_4_	0.8	3.26 F cm^–2^	0.71 mWh cm^–2^	1800
DSSC^104^	Co-NiO^*x*^	KOH	4.9	32 F g^–1^	2.3 Wh kg^–1^	270
DSSC^40^	TiO_2_	Li_2_SO_4_	3.17	∼1.1 mF cm^–2^	0.0667 μWh cm^–2^	∼18
DSSC^39^	PEDOT	LiClO_4_/MPN	4.37	0.52 F cm^–2^	-	150
DSSC^105^	AC	Pyr_14_TFSI	2.25	1.8 mAh	-	∼1.75 h
DSSC^106^	CoSbS/MWCNT	Na_2_SO_4_	6.0	382 C g^–1^	-	∼125
DSSC/QDSC^65^	PEDOP/MnO_2_	PMMA/BMIMOTf/PC	6.11	183 F g^–1^	13.2 Wh kg^–1^	132
QDSC^76^	PEDOT	HEMIMBF_4_ /PVP	-	0.667 mF cm^–2^	36.3 μWh cm^–2^	55
QDSC^41^	Ni/C	Na_2_S/S/KCl	1.83	487.8 F g^–1^	56.4 μJ	140
QDSC^78^	NiCo-MOF	KOH	2.75	134 F g^–1^	24 Wh kg^–1^	175
QDSC^42^	CNTs	PMMA/LiOTf/PC	3.45	150 F g^–1^	-	75
QDSC^77^	AC	KOH	3.94	132.83 mF cm^–2^	23.9 mJ cm^–2^	>120
*i*OPV^107^	GO:PEDOT:PSS	H_3_PO_4_/PVA	1.01	2.81 mF cm^–2^	0.18 μWh cm^–2^	∼120
OPV^79^	CNTs	H_3_PO_4_	1.1	77 μF cm^–2^	0.16 μWh cm^–2^	∼310
OPV^85^	graphene	TEABF_4_/PC	1.6	2.5 mF cm^–2^	0.2 J g^–1^	240
OPV^108^	CNTs	NaCl	1.8	3.0 mF cm^–2^	-	400 min
OPV^86^	Ti_3_C_2_T_*x*_	TT/PEGDA/EMIMTFSI	2.5	410 F cm^–3^	-	∼320
OPV^44^	CNTs	H_3_PO_4_/PVA	3.39	28 F g^–1^	2.96 Wh kg^–1^	35
OPV^109^	PEDOT:PSS	H_3_PO_4_/PVA	3.44	30 mF cm^–2^	2.57 μWh cm^–2^	25
OPV^110^	PEDOT:PSS/Ti_3_C_2_T_*x*_	H_2_SO_4_	6.7	93 mF cm^–2^	28.7 μWh cm^–2^	250
OPV^98^	RGO	H_3_PO_4_/PVA	7.85	144 F g^–1^	-	∼110
OPV^84^	PEDOT:PSS/CNTs	H_2_SO_4_/PVA	9.75	250 mF cm^–2^	-	183
OPV^81^	MPNC	H_2_SO_4_/PVA	14.0	145 mF cm^–2^	160 μWh cm^–2^	500
perovskite PV^93^	RZCo//PyR/PEDOT:PSS	LiClO_4_/AN	0.46	150 mF cm^–2^	-	∼25
perovskite PV^111^	PANI/CNTs	H_2_SO_4_ /PVA	2.7	422 mF cm^–2^	-	275
perovskite PV^112^	Co_9_S_8_–MnO_2_	H_3_PO_3_ /PVA	5.6	262.5 mF cm^–2^	-	38
perovskite PV^91^	AC/MnO_2_	LiCl/PVA	7.79	61.01 mF cm^–2^	-	>500
perovskite PV^88^	AC	H_3_PO_4_/PVA	8.9	17.5 mF cm^–2^	2.12 μWh cm^–2^	44
perovskite PV^113^	AC/RGO–PEDOT	H_2_SO_4_/PVA	11	71.7 mF cm^–2^	32.3 μWh cm^–2^	400
perovskite PV^47^	N-MC	H_2_SO_4_/PVA	12.5	31 mF cm^–2^	10.4 μWh cm^–2^	75
perovskite PV^98^	RGO	H_3_PO_4_ /PVA	13.66	142 F g^–1^	-	130
perovskite PV^97^	TiO_2_ nanotube	PVA/LiCl	13.98	3 mF cm^–2^	0.40 μWh cm^–2^	60
perovskite PV^96^	AC	KOH/PVA	14.4	13.6 F g^–1^	-	20
perovskite PV^95^	AC	KOH	22.44	123.8 mF cm^–2^	20.9 μWh cm^–2^	211.5

In addition to the PCE, the total efficiency of a PSC device highly depends on the capacitance of the SC unit. The contributions of the Helmholtz double layer are used to determine the capacitance of a charged electrode. Equation 5 shows the determination of *C*_H_ and *C*_diff_, which represent the inner layer and diffusive layer capacitance values, respectively.
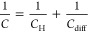
5

The specific capacitance of the SC unit can be calculated from three electrochemical processes: (i) galvanostatic charge–discharge measurements (GCD), (ii) cyclic voltammetry (CV), and (iii) electrochemical impedance spectroscopy (EIS) using eqs 6–8), respectively.
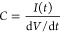
6

In eq 6, “*I(t)*” signifies the constant current applied during the GCD measurement, while d*V*/d*t* corresponds to the slope of the GCD.
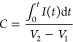
7

In eq 7, *C* is determined by integrating the *I–V* voltammogram divided by the potential window.
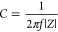
8From EIS analysis, the capacitance can be determined from the frequency and *Z*, the real part of the impedance, through eq 8. For the symmetric solid-state SC devices composed of electrodes with equal mass loading of the same active material, the capacitance of a single electrode can be determined as described in eq 9.
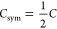
9Apart from the capacitance, the output energy (*E*_storage_), the energy density (*E*_A_) normalized by the mass loading in (Wh g^–1^) or normalized by the active area (Wh cm^–3^), and the power density (*P*_A_) (W g^1–^ or Wh cm^–3^) are two important performance criteria for SCs during the GCD process (eqs 10–12).
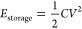
10
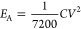
11
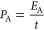
12

### 2.1 DSSC-Integrated PSCs

The first reported solar-light-charged SC device in the literature was a two-electrode electrochemical cell constructed with a redox-free liquid electrolyte sandwiched between a photoelectrode and a counter electrode.^37^ A porous activated carbon (AC) was used to make a heterojunction with both electrodes for enhanced charge storage. The Ru-complex dye-adsorbed mesoporous TiO_2_ semiconductor nanoparticles allow the transition of photogenerated electrons to the conduction band. The photogenerated carriers accumulated charge on the porous AC and achieved photocharging with a specific capacitance of 0.69 F cm^–2^ at a voltage of 0.45 V. More importantly, this result indicated the feasibility of solar charging an SC, which led to substantial attention on PSC devices. The same research group reported the three-electrode DSSC-integrated PSC device a year later, which reduced the internal resistance in a two-electrode PSC to extend the discharge time.^60^ The three-electrode PSC resulted in an areal energy density five times higher than the two-electrode PSC device reported in the earlier work. This outcome showed that the three-electrode device with a common electrode consisting of a platinum (Pt) and a carbon (C) electrode established the hole–electron transition in the charge–discharge process.

**Figure 1:**
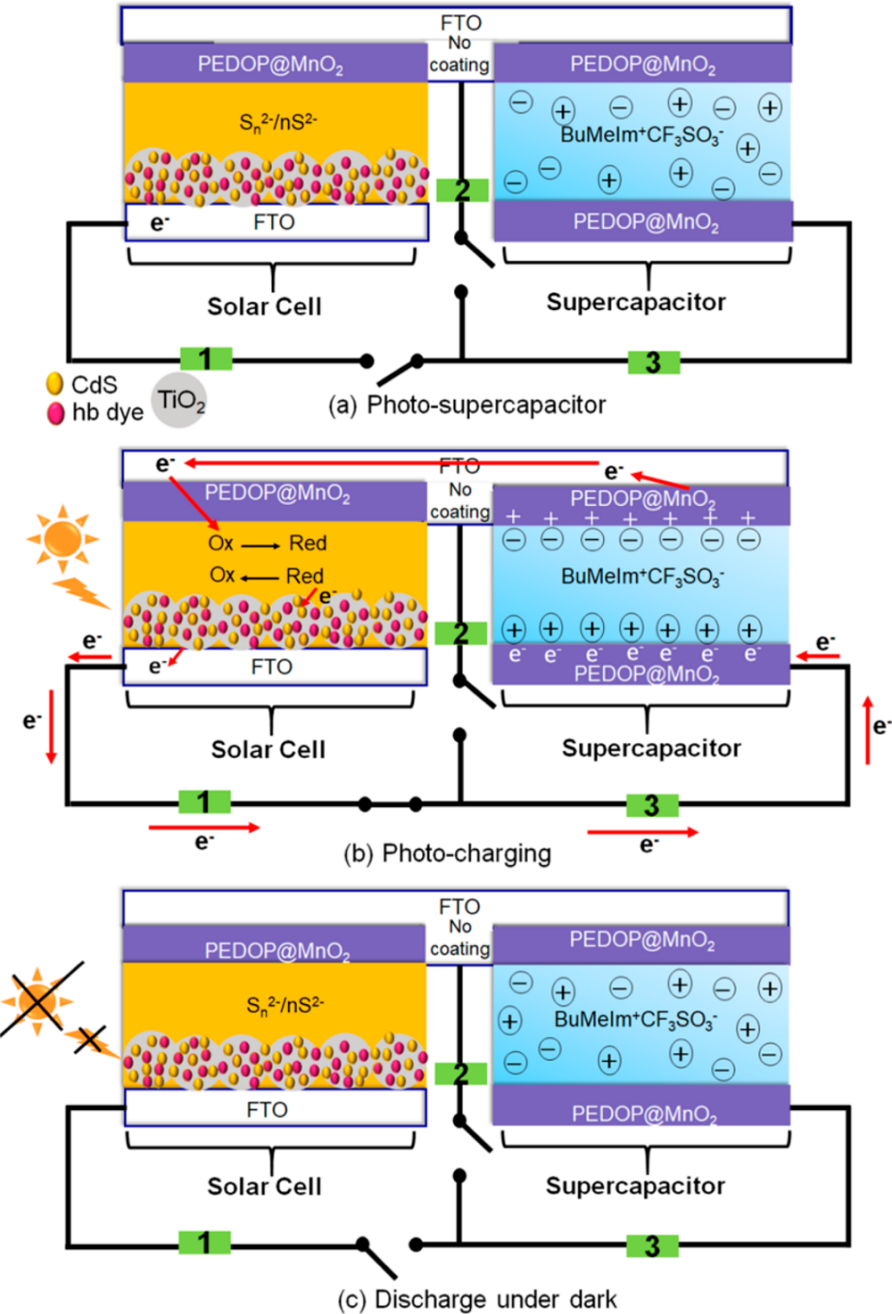
Schematics of the DSSC-integrated PSC prepared from a cadmium sulfide (CdS) quantum dots/hibiscus dye-cosensitized TiO_2_-based DSSC and poly(3,4-ethylenedioxypyrrole)@MnO_2_-based SC in the (a) as-fabricated state, (b) photocharging state, and (c) discharge state in the dark. Reproduced with permission from ref (65). Copyright 2018 American Chemical Society.

After the first demonstration, many other research works contributed to new results in the field on the basis of DSSC-integrated charge storage devices. For instance, an integrated, flexible PSC device consisting of a TiO_2_ nanotube-based DSSC and a graphene-based electrical double-layer capacitor exhibited a storage efficiency of 1.02% under 1 sun illumination.^61^ DSSC integration to laser-induced graphene as a charge storage counter electrode has been reported to build a self-charged flexible device under solar illumination.^62^ Lau et al. reported a three-electrode assembly of an integrated PSC where a graphene-based bifunctional electrode was used as a charge storage counterpart.^63^ This device obtained a specific capacitance of 124.7 F g^–1^ with a cycling stability of 70.9% after 50 consecutive charge/discharge cycles. MnO_2_ is a widely used SC material because of its pseudocapacitive nature. Therefore, an integrated device based on TiO_2_/FTO as the photoanode and manganese dioxide (MnO_2_)-coated microarray carbon nanotubes (CNTs) as the counter electrode resulted in a high discharge capacitance of 13 mF cm^–2^ at a potential of 0.932 V under 1 sun illumination.^64^ Das et al. reported a PSC device with the integration of a cadmium sulfide (CdS) quantum dots/hibiscus dye-cosensitized TiO_2_-based DSSC and poly(3,4-ethylenedioxypyrrole)@MnO_2_-based SC (Figure 1) with a specific capacitance of 183 F g^–1^ and an energy density of 13.2 Wh kg^–1^ at a discharge current density of 1 A g^–1^.^65^ The mechanisms for the photocharging and discharging in the dark are given in Figure 1. Liu et al. reported an integrated PSC device of DSSC and G/CNTs/polyaniline (PANI)-based SC as the common electrode with a storage efficiency as high as 2.1%.^66^

Song et al. constructed a flexible, lightweight PSC device with fiber-shaped DSSC and triboelectric nanogenerators promising cutting-edge wearable electronics.^67^ Recently, the monomer photovoltaic, electrochromic SCs device, by integrating DSSC and electrochromic SCs (ESCs) based on the WS_2_–WO_3_ counter electrode, was reported to achieve a specific capacitance of 69.9 mF cm^–2^ and 91.89% cyclic stability over 2000 cycles.^68^ Another example of the DSSC-integrated PSC device was constructed by utilization of a solid-state hybrid SC based on a bismuth–graphitic carbon nitride (Bi-*g*-C_3_N_4_) nanocomposite and AC electrodes as an energy storage component and TiO_2_/N_719_/I^–^/I_3_^–^/Pt-based DSSC as the solar energy converter part.^69^ The proposed device was assembled by using PVA–KOH as a gel polymer electrolyte and delivered 31.597 μWh cm^–2^ and 3.25 mW cm^–2^ for energy and power density, respectively.

### 2.2 Quantum Dot-Sensitized Solar Cell (QDSC)-Integrated PSCs

QDSCs employ metal oxide semiconductors sensitized by quantum dots attached instead of the dyes of DSSCs. These quantum dots rely on the benefits of tunable optoelectronic properties because of the adjustable size and shapes.^70,71^ The semiconductors for electrode materials used in QDSC and SC applications are usually common.^72−75^ In addition to individual research on energy conversion/storage applications, the integration of QDSCs and SCs to build up PSC devices has been reported in the literature.^42,76,77^

**Figure 2:**
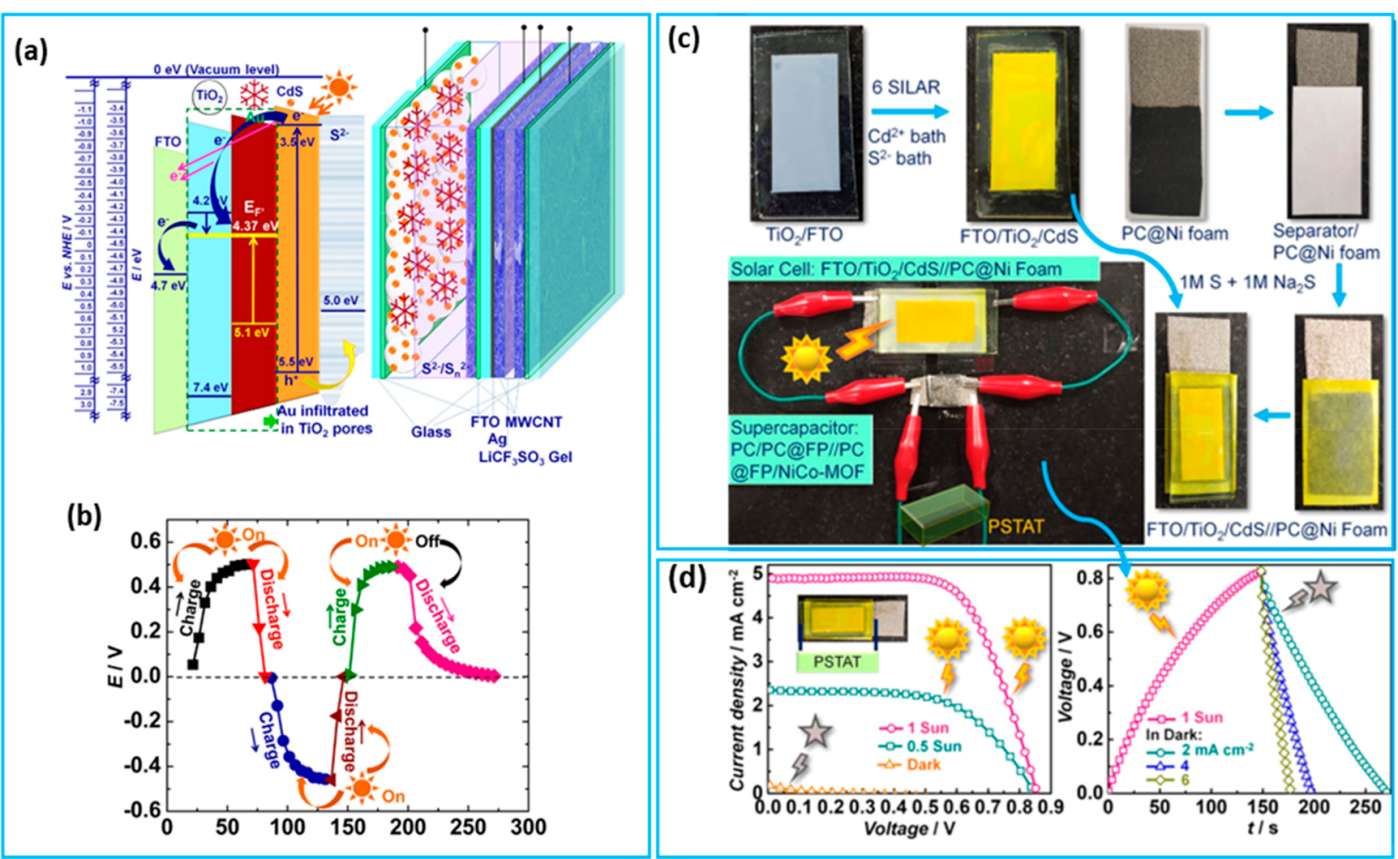
Integrated device proposed by Narayanan et al reproduced with permission from ref (42); copyright 2015 Elsevier: (a) energy band diagram of a plasmonic solar cell and a schematic of the PSC device and (b) GCD profile of the PSC device, under illumination (0.1 mW cm^–2^) and in the dark. QDSC-integrated device reported by Ohja et al. reproduced with permission from ref (78); copyright 2020 American Chemical Society: (c) fabrication process of a solar cell-integrated PSC device and (d) *J*–*V* characteristics of the solar cell in the dark and under 0.5 and 1 sun conditions, photocharging of the PSC under 1 sun illumination, and GCD at different current densities in the dark.

QDSC-integrated PSC devices were first reported by the preparation of the device on the basis of a TiO_2_/CdS/Au electrode as the photoanode and a SC part comprising an intermediate electrode of MWCNT/FTO/glass/Ag/MWCNT (MWCNT, multiwalled carbon nanotube; FTO, fluorine-doped tin oxide) and a counter electrode of MWCNT/FTO.^42^ This device exhibited 150 F g^–1^ for the capacitance of the symmetric SC, with no external bias under illumination with 0.1 mWcm^-2^ power density (Figure 2a,b). The planar solid-state PSC device was constructed as a two-electrode configuration of ZnO nanorods/ZnS/Ag_2_S quantum dots for the photoanode material. Conversely, poly(3,4-ethylene-dioxythiophene) (PEDOT) film was used for the SC.^76^ Consequently, this device produced a storage efficiency of 6.83% at a potential of 0.33 V during photocharge. Zheng et al. reported research on the basis of a CdS/CdSe QD-cosensitized solar cell and an active carbon-based SC with a shared electrode and separate aqueous electrolytes.

This device achieved an areal capacitance of 132.83 mF cm^–2^ and an energy density of 23.9 mJ cm^–2^.^77^ Shi et al. prepared a CdS/CdSe QDs-cosensitized mesoporous TiO_2_ solar cell for integration with a PSC device consisting of an asymmetric SC electrode of Cu_2_S and carbon films.^41^ This device takes advantage of the uses of monoelectrolyte and the shared double-sided middle electrode to avoid separate sealing problems. Another CdS-based QDSC utilized for integrating the PSC device was built with asymmetric SCs consisting of a porous carbon (PC) and a NiCo-MOF (metal–organic framework) as anode and a cathode, respectively (Figure 2c,d).^78^ This device delivered a capacitance of 520 F g^–1^ and an energy density of 92 Wh kg^–1^.

### 2.3 Organic PV (OPV)-Integrated PSCs

**Figure 3:**
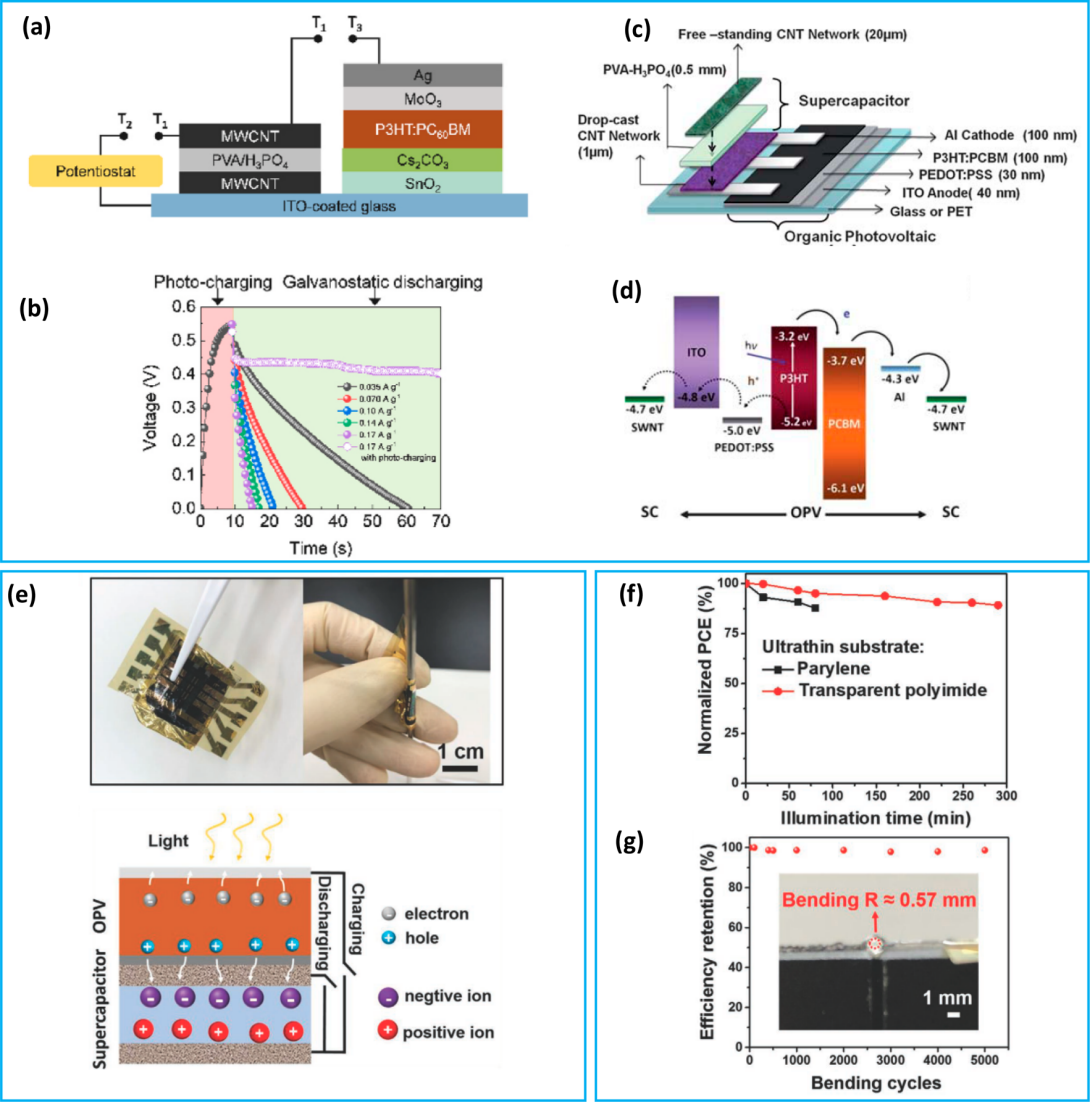
(a) Schematic representation for the OPV-integrated PSC device; SC (left) and OPV (right). (b) Galvanostatic discharge profiles of the IPS at different currents after being photocharged. Reproduced with permission from ref (83). Copyright 2021 Elsevier. (c) A schematic representation of a printable OPV-integrated PSC device on a common platform and (d) circuit illustration during the charging process. Reproduced with permission from ref (44). Copyright 2011 Royal Society of Chemistry. (e) Photographs of the fabricated ultrathin device and schematic working mechanism, (f) stability of the PSC device under light illumination, and (g) bending results of the OPVs over 5000 cycles. Reproduced with permission from ref (84). Copyright 2020 Wiley-VCH.

OPV devices grant benefits of being low cost, lightweight, and flexible among other PV devices. Thus, several studies have been reported on the basis of OPV-integrated PSC systems.^79−82^ Shin et al. reported an integrated PSC device utilizing the organic PV and solid-state SC in which both share an indium–tin-oxide (ITO) electrode for enhanced charge propagation (Figure 3a,b).^83^ The overall energy conversion–storage efficiency of 2.27% was achieved when the device was charged under 1 sun illumination. In another work, single-walled carbon nanotube (CNT) networks and OPV devices were printed on a common platform to build a printable OPV-integrated PSC device (Figure 3c,d).^44^ Liu et al. reported a flexible and ultrathin planar device based on OPV.^84^ The photoanode was an atomically thin polyimide platform bearing ZnO as the electron transport layer, a bulk heterojunction polymer with [6,6]-phenyl-C71-butyric acid methyl ester (PC71BM) as the photosensitizer, MoO_*x*_ as a hole transport layer, and Ag as the top contact for improved passivation. This device exhibited cycling stability lasting 1 week (100 cyclic photocharge–discharge cycle) under 100 mW cm^–2^ light illumination and endured over 5000 bending cycles.

Chien et al. developed a power system combining OPV on the basis of poly(3-hexylthiophene)(P3HT)/phenyl-C61-butyric acid methyl ester (PC_60_BM) bulk heterojunction cells with aluminum electrodes and graphene-based SC, which provided 5 V of open-circuit voltage.^85^ A monolithically integrated device of high-performance OPV with mesoporous nitrogen-doped carbon nanosphere-based SCs was constructed in a three-electrode configuration and resulted in 17% photoelectrochemical conversion efficiency.^81^ Ti_3_C_2_T_*x*_-type MXene has been utilized as a transparent common electrode in a PSC device by integrating a flexible OPV to display a high volumetric capacitance (502 F cm^–3^) and a high power conversion efficiency of 13.6%.^86^ Another PSC device fabricated using a dual-functional-layered graphene oxide (GO)-incorporated PEDOT:PSS as the common electrode exhibited ∼81% energy storage efficiency.^87^

### 2.4 Perovskite PV-Integrated PSCs

**Figure 4:**
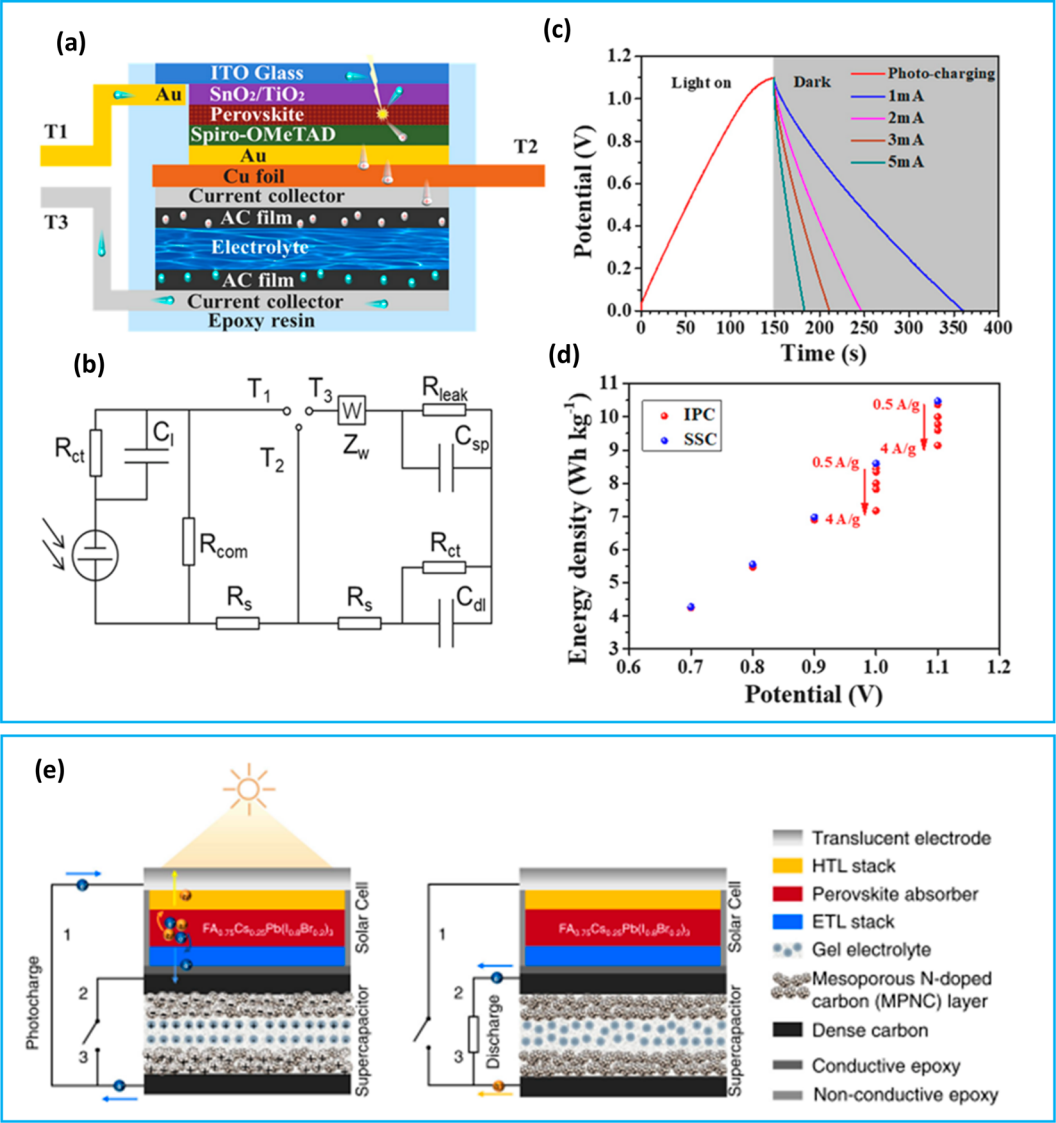
(a) Structural illustration of the structure, (b) equivalent circuit, (c) photocharging/galvanostatic discharge (at various current densities) profile, and (d) energy densities at different voltage windows in GCD for perovskite-integrated PSC device reported by Song et al. Reproduced with permission from ref (95). Copyright 2022 Elsevier. (e) Schematic diagram of the PSC architecture fabricated via vertical integration of the SC unit with the PV cell through a shared carbon electrode connection at photocharging mode (on the left) and dark discharging mode (on the right). Reproduced with permission from ref (47). Copyright 2021 The Authors; published by Wiley-VCH GmbH.

Perovskite solar cells have displayed remarkable performance for energy conversion/storage applications over the past few years because of their incomparable optoelectronic properties, such as high charge carrier mobility, large absorption coefficient, and regulable band gap values.^88−94^ Liang et al. employed perovskite solar cells for a self-charging energy device with an energy conversion efficiency as high as 7.1% in the photocharging mode.^88^ A photovoltachromic cell was constructed by integrating a perovskite solar cell (PSC) and MoO_3_/Au/MoO_3_ transparent electrode and electrochromic SC to provide in situ energy storage.^92^ Another PSC device built as a three-terminal photo capacitor by integrating a perovskite solar cell and symmetrical SC was reported to have 20.5% solar energy conversion storage efficiency.^95^Figure 4a,b presents the PSC device consisting of active carbon (AC) paste layers symmetrically assembled (AC//KOH//AC) using 6 M KOH and a planar perovskite solar cell (PSC) with the configuration of ITO/SnO_2_/TiO_2_/FAMAPb(IBr)_3_/spiro-OMeTAD/Au and proposed equivalent circuit (EC). Upon the photocharging under 1 sun illumination, potential increases to the open circuit potential of the PSC device (∼1.1 V) and various dark discharge times at current densities ranging from 1 to 5 mA were observed (Figure 4c). The integrated device has an energy density of 10.17 Wh kg^–1^ (20.34 μWh cm^–2^) at 1.1 V of the potential window (Figure 4d). In Berestok et al.’s work (Figure 4e), a FA_0.75_Cs_0.25_Pb(I_0.8_Br_0.2_)_3_ perovskite solar cell-integrated PSC device with a gel electrolyte-type SC composed of N-doped carbon nanospheres exhibited photoelectrochemical energy conversion efficiency of 11.5% and SC storage efficiency of 92%.^47^

Zhang et al. reported an integrated hybrid power pack consisting of a perovskite solar cell (PSC) and SC.^97^ In the system, double-sided TiO_2_ nanotubes were employed in dual functions as an ETL for PSC and a cathode for SC, respectively. Compact and monolithically stacked self-charging power packs prepared by integrating an organometal halide perovskite- or polymer-based solar cell and SC in a single device exhibited a very high storage efficiency (η_storage_) of 80.31%.^98^ PSCs–OSCs tandem solar cells have been integrated into solid-state asymmetric SC devices to build wireless, portable, lightweight self-charging power packs and have displayed a high overall efficiency of 12.43% and an energy storage efficiency of 72.4% under white light illumination.^45^ A lead-free perovskite-based PV device prepared from Cu_3_Bi_2_I_9_ was reported for an integrated PSC by Popoola et al., which resulted in a 621 mF g^–1^ gravimetric capacitance value under illumination and exhibited a considerable retention over 10 000 cycles.^99^

## 3 All-in-One Systems

**Figure 5:**
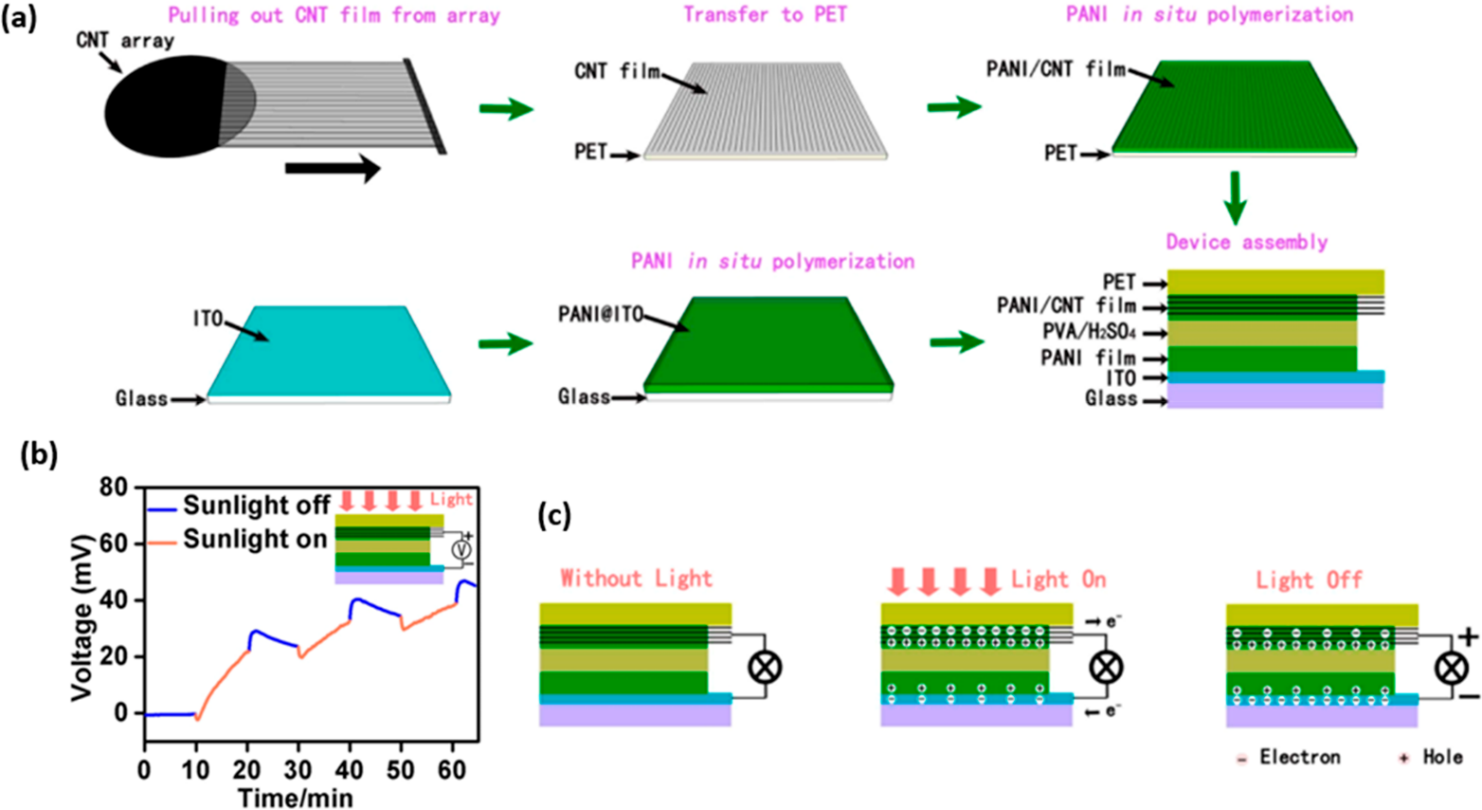
(a) Schematic illustration for the assembly process of the device, (b) the open-circuit potential with the on/off cycling (AM 1.5 solar illumination at a power density of 1000 W m^–2^), and (c) schematic illustration for the working mechanism. Reproduced with permission from ref (114). Copyright 2015 American Chemical Society.

As summarized in the previous section, photovoltaic and SC devices must be prepared separately in PSC devices to integrate with solar cells. However, dual-acting electrodes enable us to prepare all-in-one PSC systems. In this design, photoactive electrodes simultaneously provide solar energy conversion and storage. Thus, two-electrode compact systems can be fabricated. This concept is relatively new, facile, and cost-effective compared with the PV-integrated designs. One of the pioneering studies using dual-effect electrodes prepared with two electrode configurations is the PANI/CNT film published by Yin et al. and the configuration using only PANI film (Figure 5).^114^ PANI thin films are used as the photoactive and pseudocapacitive layers in this work. The PVA/H_2_SO_4_ gel was used as the solid electrolyte, and the photogenerated current density obtained was 2.0 mA g^–1^. As shown in Figure 5b, the voltage of the device increased to 48 mV after three cycles of illumination because of the photovoltaic effect of PANI as a polymer semiconductor (Figure 5c).

**Figure 6:**
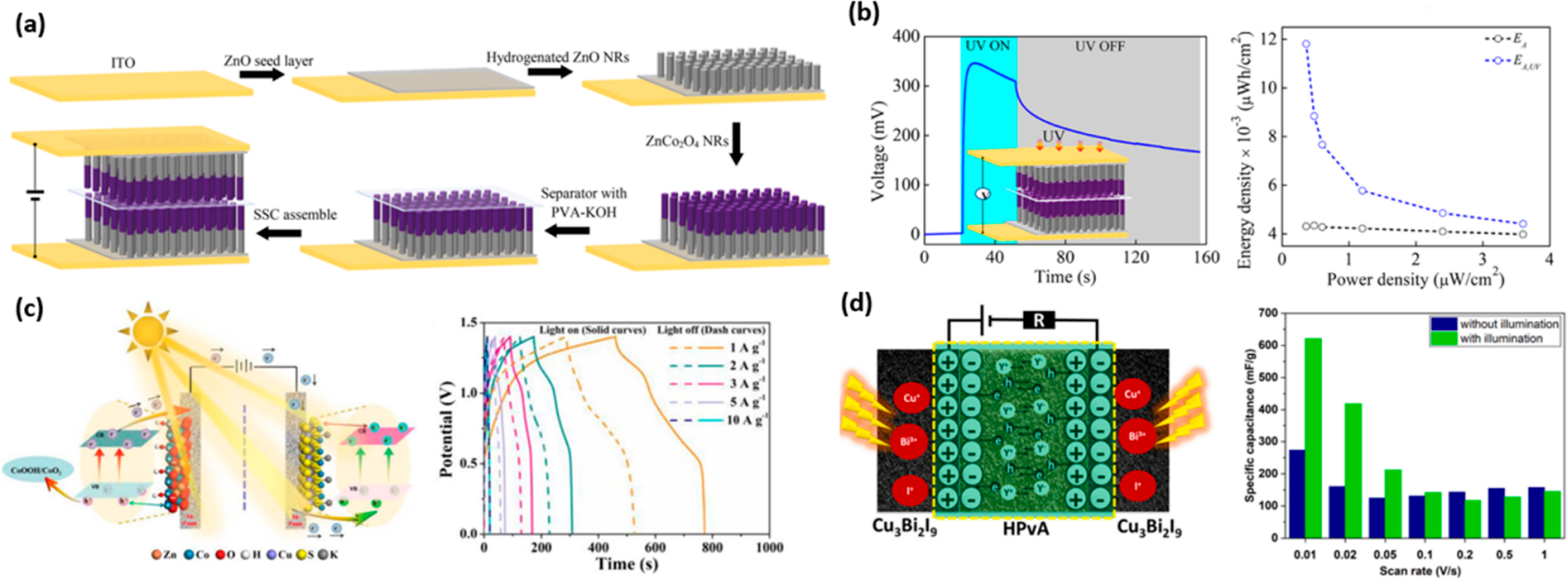
(a) Schematic illustration of fabrication processes and (b) self-generated voltage response of the self-powered PSC device under on/off UV radiation. Reproduced with permission from ref (115). Copyright 2019 American Chemical Society. (c) Photoelectrochemical charge storage mechanism of the ZCO NF//CCS HS asymmetric supercapacitor (ASC) system and GCD curves at various current densities. Reproduced with permission from ref (116). Copyright 2023 Royal Society of Chemistry. (d) Photoelectrochemical energy storage mechanism and comparison of specific capacitance as a function of scan rate of Cu perovskite-based PSC. Reproduced with permission from ref (119). Copyright 2023 Elsevier.

Metal oxides are also excellent candidates for enabling solar energy conversion and charge trapping for self-charge storage. Boruah and Misra utilized the zinc cobalt oxide and zinc oxide (ZCZO) nanorods (NRs) electrodes in an optically driven, self-powered SC in which PVA–KOH gel electrolyte was used to separate two electrodes (Figure 6a,b).^115^ In their work, the photogenerated areal capacitance and energy density under UV were reported to be 150 μF cm^–2^ and 11.8 nWh cm^–2^. Additionally, Boruah and Misra reported 350 mV of self-powered photovoltage by exposure to UV radiation. Another oxide-based dual photoelectrode, which included nanoflowerlike ZnCo_2_O_4_ (ZCO NF) as the positive electrode, was reported by Zhao et al.,^116^ who also used light-sensitive negative electrodes of hollow sphere-structured CuCo_2_S_4_ (CCS HS) (Figure 6c). That work reported that the energy density increased from 46.5 to 60.9 Wh kg^–1^ after illumination. They also reported that the charge–discharge times increased after photoirradiation (Figure 6c). Besides, the maximum specific capacitance of the ZnCo_2_O_4_//CuCo_2_S_4_-based devices was calculated as 448 F g^–1^ with photoassistance, whereas the specific capacitance under darkness was 340 F g^–1^. In their recent work, Prakash et al. reported symmetric V_2_O_5_||V_2_O_5_ and asymmetric V_2_O_5_||AC PSC devices^117^ where the asymmetric PSC device displayed an enhanced energy density from 3.6 to 9.8 Wh kg^–1^ at a power density of 29 W kg^–1^ under light illumination. In another recent study, a photocathode prepared from p–n junction α-(Fe_2_O_3_)_1–*x*_(Cr_2_O_3_)_*x*_ nanoflakes and a V-doped TiO_2_-based photoanode was assembled using semisolid PVA–KOH electrolyte to build up a PSC device with an energy density of 2.30 μWh cm^–2^ at a power density of 1.28 mW cm^–2^.^118^ Bismuth-based organometallic-halide perovskite is also among the photoactive materials for all-in-one design.^99^ Bismuth-based perovskites offer low toxicity compared with lead-based ones. One of the most critical issues of the perovskite-based PSC is the choice of electrolyte because of its moisture sensitivity. Popoola et al. used polymer CPH-G gel electrolyte to extend the cycle life of the perovskite-based PSC.^99^ In their recent study, Popoola et al. used inorganic Cu_3_Bi_2_I_9_ perovskite material to fabricate a Cu perovskite with HPvA gel electrolyte.^119^ They reported a 127% increment in specific capacitance under illumination compared with that calculated at dark (Figure 6d), and a 93.8% capacitance retention after 10 000 charge–discharge cycles was obtained for their device.

**Figure 7:**
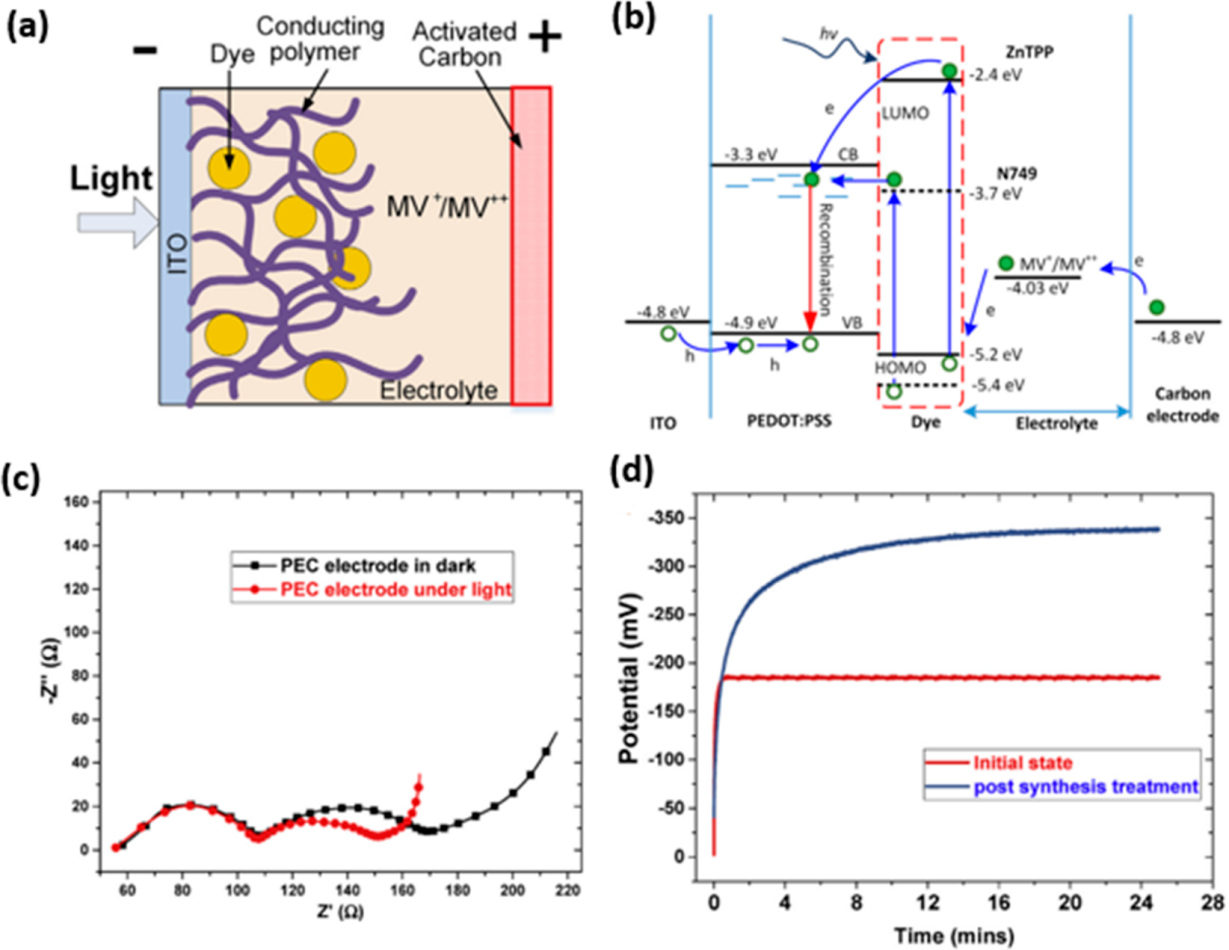
(a) Schematic of an electrochemical device with a composite of a conducting polymer and a dye as the photosensitive anode electrode and (b) energy diagram of the photoactive supercapacitor. Reproduced with permission from ref (120). Copyright 2015 Elsevier. (c) Nyquist plot obtained from EIS analysis at open-circuit potential (−50 mV vs Ag/AgCl) superimposed with a sinusoidal signal of 5 mV amplitude and 100 kHz to 1 Hz frequency range. (d) Photocharging in the illuminated open circuit condition of BiVO_4_ -RGO electrode. Reproduced with permission from ref (123). Copyright 2020 Elsevier.

Dual-acting electrodes are also used in photoelectrochemical (PEC) energy conversion/storage devices.^120−123^ Generally, PEC energy systems use liquid electrolytes. The ionic conductivity of the electrolyte solution affects the resistance and capacitance of the system, and the properties of the electrolyte, such as pH and concentration, also affect the cycle life and corrosion resistance. In their excellent review, Lv et al. summarized two- and three-electrode PEC energy storage devices, including SCs and batteries.^124^ Takshi et al. successfully demonstrated the PEC PSC system in 1 mM methyl viologen in 0.1 M Tris buffer solution where PEDOT:PSS and a porphyrin dye-coated ITO/glass substrate were used as the negative electrode (Figure 7a). As the proposed mechanism is given in Figure 7b, dye molecules generate electrons that can reduce the PEDOT:PSS. A potential difference occurs with the reduction of electrolytes by dye molecules and ion diffusion toward the counter electrode.^120^ Renani et al. reported a capacitance increase of 65% under illumination for the BVO-V_2_O_5_@TiNT electrode, which was PEC-tested in a 3 M KCl electrolyte.^121^ In their work published in 2020, Roy et al. performed the tests to determine the PEC capacitor performance of the BiVO_4_-RGO (reduced graphene oxide) bilayer electrodes.^123^ They observed decreased charge transfer resistance and junction capacitance via illumination, as evidenced by electrochemical impedance spectroscopic analysis (Figure 7c). This device generated a photovoltage of 340 mV at open-circuit conditions after keeping the PEC cell in an illuminated open-circuit condition over 16 h, which they called “postsynthesis treatment” in their work (Figure 7d).

Safshekan et al. also used BiVO_4_ as an active material in the dual-acting electrode design of PSC.^125^ They prepared BiVO_4_-PbO_*x*_ heterostructures, where BiVO_4_ served as the photoactive layer, and PbO_*x*_ provided the capacitive top layer. This device exhibited a specific capacitance of 6 mF cm^–2^ and an open cell voltage of 1.5 V vs RHE. Another oxide material, which is Cu_2_O, was also reported as a photosensitive electrode for a pseudocapacitive device.^126^ In that work, the hybrid array electrode of nanoporous Cu@Cu_2_O delivered a specific capacitance of 782 F g^–1^ at 1 A g^–1^ under illumination, which resulted in an increase of 37.9% in capacitance compared with that under dark. Wang et al. also reported a photoassisted rechargeable PEC SC in which the capacitive material, Ti_3_C_2_T_*x*_, was modified by nitrogen-doped carbon dots (NCDs) to produce light sensitivity.^127^ They reported a volumetric capacitance of 1445 F cm^–3^ (630 F g^–1^) at 10 A cm^–3^ under photoassisted charging, which is an increase of 35.9% compared with the capacitance under dark conditions.

**Figure 8:**
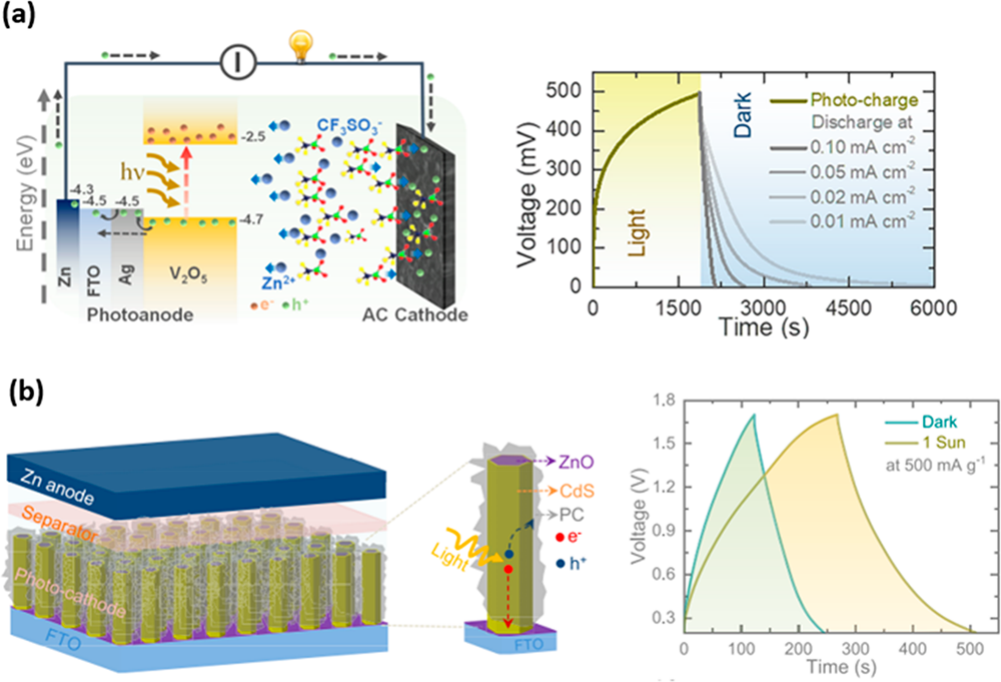
(a) Schematic representation of *h*ν-ZICs using Ag@V_2_O_5_ photoanodes and AC cathodes and photocharge (λ = ∼455 nm, *P*_in_= ∼12 mW cm^–2^) and discharge at different current densities (0.1–0.01 mA cm^–2^). Reproduced with permission from ref (132). Copyright 2020 American Chemical Society. (b) Proposed photo-ZIC device prepared from the PC/CdS@ZnO NR photocathode and Zn anode and comparative GCD curves in the dark and under 1 sun illumination. Reproduced with permission from ref (134). Copyright 2023 American Chemical Society.

Another device structure can be summarized as a separate category in two-electrode configurations where the dual-acting electrodes explained above are the hybrid systems that resemble both battery and SC structures, known as the hybrid SC that has one batterylike and one capacitorlike electrode. This design provides superior energy density and specific capacitance compared with the pseudo- or electrochemical double-layer capacitor (EDLC)-SC.^128,129^ There are reports on solar-assisted charging of lithium-ion, sodium-ion intercalation, and zinc-ion batteries.^130−142^ Among these hybrid conversion/storage systems, photorechargeable zinc-ion batteries have the potential to be safe and efficient compared with lithium-ion-type batteries. Boruah and De Volder et al. have done many pioneering studies that shed light on developing zinc-ion hybrid capacitors (ZIC) that can be charged with solar energy.^132−134,141−143^ They investigated the heterojunction-based photoactive electrodes, such as reduced graphene oxide (rGO)/g-C_3_N_4_, rGO/P_3_HT/V_2_O_5_, ZnO/MoS_2_, ZnO/VO_2,_ and CdS@ZnO NRs, for photo-ZIC applications (Figure 8). Among these works, Ag@V_2_O_5_ photoanodes utilizing photo-ZIC showed an ∼63% capacity increase under illumination.^132^ Moreover, maximum power and energy densities of 53.13 Wh kg^–1^ and 1384.61 W kg^–1^ were reported, respectively. In another work, the porous carbon (PC) coated on cadmium sulfide -decorated zinc oxide nanorod (PC/CdS@ZnO NR) array photocathode resulted in ∼99% capacity enhancement at 500 mA g^–1^ under illumination compared with dark conditions.^134^ The recent progress on all-in-one PSC devices in terms of efficiency, which is described as energy density and capacitance values, and device properties is given in Table 2.

**Table 2:** Literature Comparison of All-in-One PSC Devices

PSC device	electrolyte/separator	capacitance	current density	potential (V)	energy density
MnO_2_–V_2_O_5_/W-TiO_2_^144^	1.0 M LiCl	95 mF cm^–2^	0.12 mA cm^–2^	0.5	-
CF-MoS_2_^145^	PVA/H_2_SO_4_	7 mF cm^–2^	0.01 mA cm^–2^	-	-
N-MCN@GH^146^	PVA/H_2_SO_4_	8.1 F cm^–3^	1.0 A g^–1^	1.0	1.12 mWh cm^–3^
V_2_O_5_||AC^117^	K_2_SO_4_–PVA	120 mC cm^–2^	0.1 A g^–1^	1.8	3.6 Wh kg^–1^
Ni(OH)_2_/TiO_2_^52^	1 M NaOH	22.9 mF cm^–2^	0.05 mA cm^–2^	0.45	-
MAIBiI_3_^99^	PVA/CB-H_3_PO_4_	4.0 mF cm^–2^	0.01 mA cm^–2^	1.0	0.55 μWh cm^–2^
BiVO_4_–PbO_*x*_^125^	0.1 M phosphate buffer	4.5 mF cm^–2^	0.015 mA cm^–2^	1.5	-
FTO/α-Fe_2_O_3_/Ni(OH)_2_/PW^147^	1 M KCl	2.16 mF cm^–2^	0.12 mA cm^–2^	1.0	-
V-TiO_2_/α-(Fe_2_O_3_)_1-*x*_(Cr_2_O_3_)_*x*_^118^	KOH–PVA	6.5 mF cm^–2^	0.33 mA cm^–2^	1.6	2.30 μWh cm^–2^
CNT@TiC/CNT@TiO_2_^148^	PVA/LiCl	7.35 mF cm^–2^	0.20 μA cm^–2^	0.3	-
Bi_2_S_3_/MWCNT^149^	Na_2_SO_4_/PU foam	48 F g^–1^		1.8	10.4 Wh kg^–1^
V_2_O_5_/ZnO^150^	PVA–KCl/filter paper	20 mF g^–1^	0.20 μA cm^–2^	0.8	-
g-C_3_N_4_/ZnO NW^53^	PVA–LiCl/BPS-Li boiled	3.6 F g^–1^	53 mA g^–1^	2.2	8.65 Wh kg^–1^
GO/ZnO NW^54^	PVA–LiCl/filter paper	7.2 F g^–1^	0.20 A g^–1^	2.5	22.50 Wh kg^–1^
rGO/ZnO NW^151^	PVA–LiCl/filter paper	875 mF g^–1^	28.00 mA g^–1^	1.5	0.10 Wh kg^–1^
MnS@V_2_O_5_–BiVO_4_^152^	PVA–KCl	41.6 F g^–1^	10 μA cm^–2^	1.5	7 Wh kg^–1^

## 4 Defect Engineering

One of the strategies to improve photoactivity and electrochemical energy storage performance, such as optical response, recombination process, energy density, power density, and cycle life spans, is through defect engineering.^153^ The defects can disturb the neighboring atoms somewhat and cause lattice distortion in the crystal materials, thereby modifying the electronic structure and chemical properties to optimize the electrochemical properties. Also, they can provide more active sites, which accelerates ionic transfer and stabilizes the cathode structure. Generally, the defects are classified in function of their dimensions into the point defects 0D (vacancies and heteroatomic doping), line defects 1D (dislocation), planar defects 2D (grain and twin boundary), and volume defects 3D (void, amorphous, and disorder).^154−156^ The following describes two types of defects frequently generated in materials used in photoenergy storage applications: point defects and planar defects.

### 4.1 Point Defects

Intrinsic defects and extrinsic defects representative of 0D point defects have been reported to decrease the activation energy of electrochemically active species reaction; also, they own a great number of localized electrons, which act as functional sites capable of adsorbing guest ions for enhancing the specific capacity of the host materials.^157^

#### 4.1.1 Intrinsic Defects

The thermal vibration of lattice atoms generates intrinsic defects, such as vacancies, without modification of the crystal material composition. Vacancy formation generates an interstitial defect at the new location or the disappearance of the oppositely charged ions.^158^ The anionic O and S vacancies have been reported to improve different materials’ photoactivities and electrochemical performance. Xu et al. obtained an electrode material on the basis of defective titanium oxide nanotubes for dye-sensitized solar cells (DSSC) and electrochemical SCs. SC performance was enhanced by selective plasma-assisted hydrogenation treatment inducing Ti^3+^–O vacancies, which increased the electrical conductivity and charge carrier density.^159^ The optimized PSC device exhibited a remarkable overall photoelectric conversion and storage efficiency of up to 1.64%, with fast response and superior cycling capability for more than 100 photocharge/galvanostatic discharge cycles without decay.^40^ Recently, Cui et al.^153^ developed birnessite-MnO_2_ with oxygen vacancies by combining the mild H- and O-plasma.

**Figure 9:**
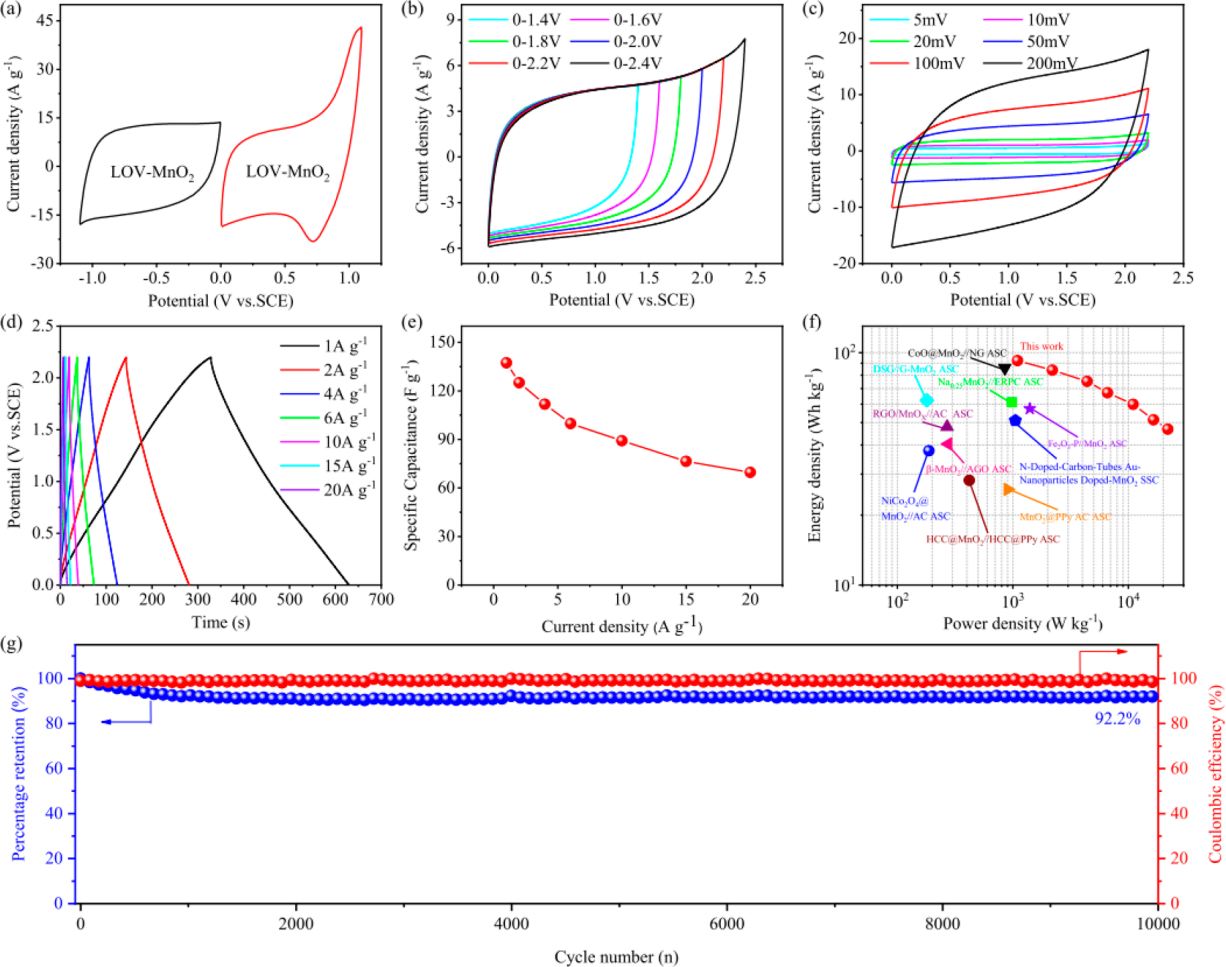
Electrochemical performance of the configured DSSC device: (a) CV curves at a scan rate of 50 mV s^–1^ of LOV-MnO_2_, (b) CV curves of the LOV-MnO_2_//LOV-MnO_2_ in different voltage windows (scan rate: 50 mV s^–1^), (c) CV curves at varied scan rates of LOV-MnO_2_//LOV-MnO_2_, (d) GCD curves at different current densities of LOV-MnO_2_//LOV-MnO_2_, (e) Specific capacitance values measured under different current densities of LOV-MnO_2_//LOV-MnO_2_, and (f) Ragone plot. The maximum energy densities at specific power densities of other symmetric and asymmetric SCs reported in the literature are provided for comparison. (g) Cycle performance and Coulombic efficiency at 20 A g^–1^ for 10 000 cycles. Reproduced with permission from ref (153). Copyright 2021 Elsevier.

As presented in Figure 9, the sample with stable oxygen vacancies in the lattice has the highest electrochemical performance, e.g., specific capacitance as high as 445.1 F g^–1^ (at a current density of 1.0 A g^–1^) with a capacitance retention of 96.6%. Besides, the configured symmetrical SC device of LOV-MnO_2_//LOV-MnO_2_ (LOV-MnO_2_, lattice oxygen vacancies in birnessite-MnO_2_) delivers an energy density of 92.3 Wh kg^–1^ at a power density of 1100.3 W kg^–1^ with a widened working voltage of 2.2 V. An outstanding cyclic life of 92.2% capacitance retention was also achieved after 10 000 charge–discharge cycles. The electrochemical performance enhancement was explained on the basis of the effect of oxygen vacancies. Oxygen vacancies reduce the valence state of Mn^4+^ to Mn^3+^ ions, which is beneficial for the electrode conductivity because of the existence of different valence states of Mn and facilitates the electrolyte ions’ diffusion kinetics. Also, the electrochemical performance of transition metal dichalcogenides was improved by introducing sulfur vacancies, which affect the electronic structure. Liu et al. obtained a series of sulfur-deficient TiS_2_ with anion-controlled concentration by adjusting annealing temperatures and stoichiometric ratio (Ti/TiS_2_). It was demonstrated that the introduction of sulfur vacancies benefits the stability of the structure by increasing the Ti–S bond strength and the electronic conductivity. Hence, the electrochemical characteristics of TiS_2_ are greatly optimized, including cycle ability and dynamic characteristics.^155^

#### 4.1.2 Extrinsic Defects

**Figure 10:**
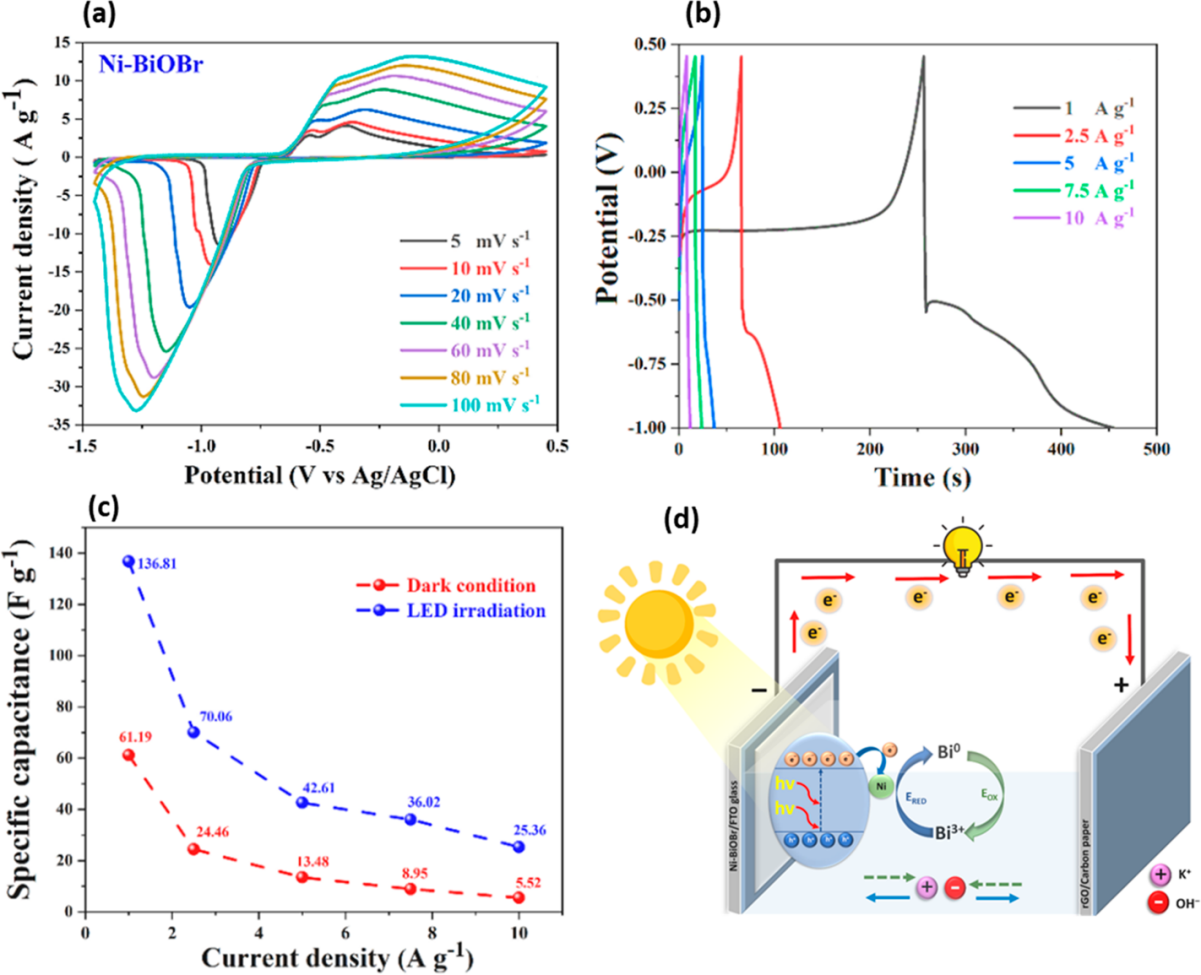
CV curves of Ni-BiOBr//rGO ASC device (a) under LED conditions at various scan rates and (b) under LED conditions at various current densities, (c) the calculated specific capacitance by GCD at various current densities under LED and dark conditions, and (d) a schematic of the Ni-BiOBr//rGO ACS device. Reproduced with permission from ref (164). Copyright 2023 Elsevier.

Extrinsic defects are due to external atoms or ions substituting original atoms in a crystal lattice or entering interstitial positions, which results in local lattice distortion.^160^ Cation or anion doping effectively narrows the band gap energy and extends the optical response to the visible absorption range, thereby enhancing the separation of the photogenerated electron–hole pairs and photocatalytic efficiency.^161^ Besides, the insertion of doping atoms/ions in the crystal lattice also provides multiple redox reactions that enhance the electrolyte diffusion into the crystal lattice, which is favorable to improving the electrocapacitance properties.^162,163^ Khampunbut et al. developed Ni-doped BiOBr nanosheets for photoassisted charging of SCs by utilizing solar energy to enhance the storage capacity.^164^ It was shown that the presence of Ni ions increased the electrochemical performance by forming oxygen vacancies in BiOBr, which facilitated fast ion/charge transportation. Moreover, Ni doping narrows the BiOBr band gap, thereby allowing electrons to participate in charge storage processes. The mechanism of photoassisted charge storage for Ni-BiOBr was elucidated and explained. Under visible light, the Ni-BiOBr electrodes generate e^–^–h^+^ pairs needed in electron storage and redox reaction; the electrical current is stored through the reduction of the anode surface (Bi^3+^ to Bi^0^), as well as electrostatic absorption of K^+^ ion. The discharging process takes place in the inverse process: (i) electrons are released by the photogenerated holes in the VB of BiOBr and transferred to the external circuit, and (ii) the electrical storage is simultaneously released through the oxidation at the Ni-BiOBr surface by oxidizing the Bi^0^ to Bi^3+^along with the electrostatic desorption of K^+^ ion (Figure 10).

### 4.2 2D Planar Defects

2D planar defects are acquired by constructing heterostructures by combining various semiconductor materials. The electrons can move in a single direction at the heterostructure interface to forming the built-in electric fields at the 2D heterointerface.^165^ A heterostructure’s main advantage is a better separation of photogenerated charges, which can promote electrochemical storage.^166^ Zhao et al. developed a photoresponsive heterogeneous junction between NiO and FeCo_2_O_4_ (FCO) with improved performance for photoassisted SC.^167^ The obtained electrodes exhibit an ultrahigh energy storage capacity of 7933 F g^–1^ at 1.0 A g^–1^ under simulated sunlight.

**Figure 11:**
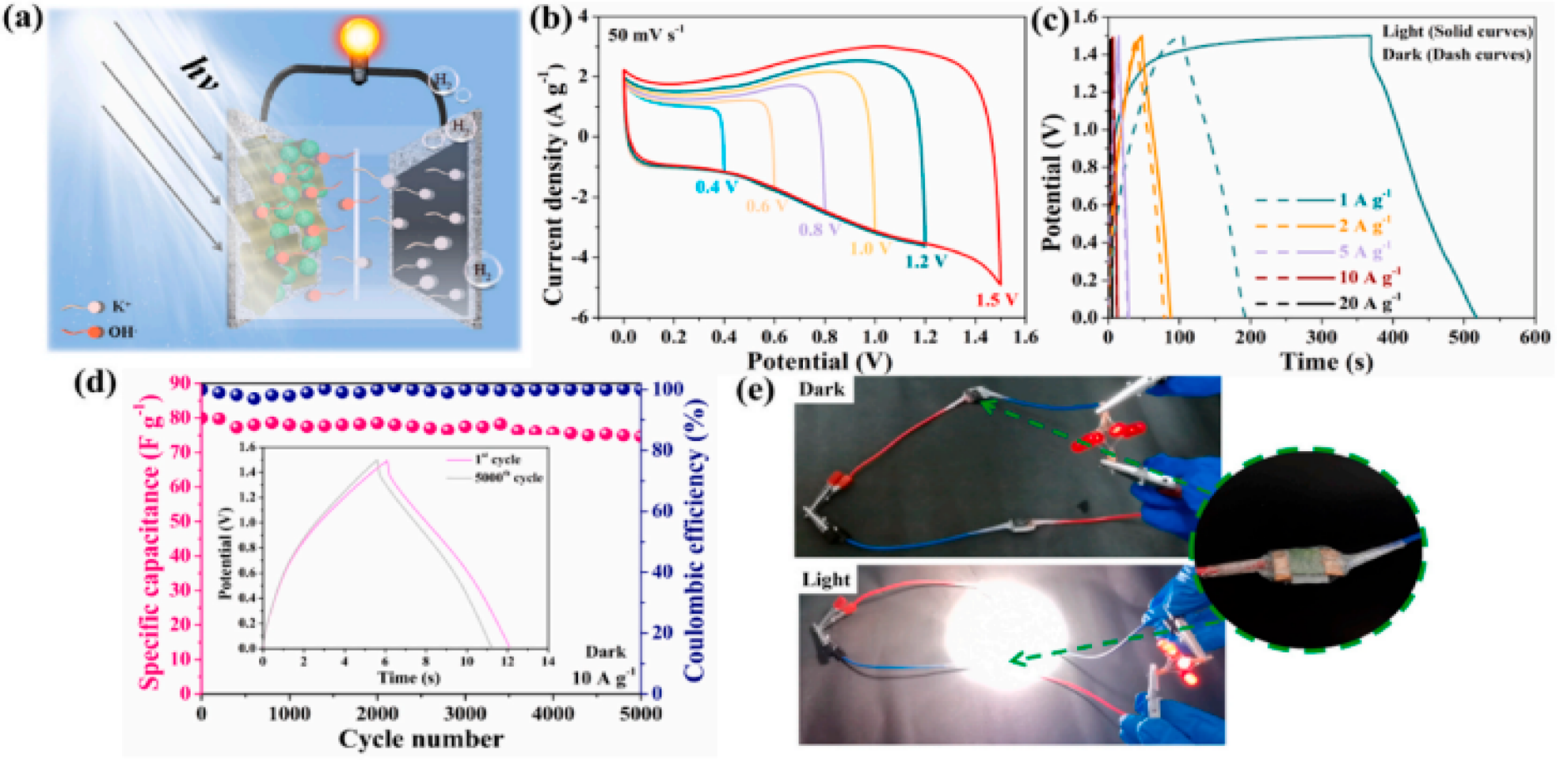
(a) Schematic illustration of the NiO/FCO//AC ASC device, (b) CV curves of the ASC with the increase of the potential window, (c) GCD curves of the ASC at different current densities under dark (dash curves) or light (solid curves), (d) cycling stability and Coulombic efficiency of the ASC at a current density of 10 A g^–1^ under dark with an inset showing the GCD of the ASC at a current density of 10 A g^–1^ after the 1st and 5000th cycles under dark, and (e) the photos of the LEDs lit by the ASC under dark (up) or light (down). Reproduced with permission from ref (167). Copyright 2022 Elsevier.

In addition, a NiO/FeCo_2_O_4_//AC asymmetric SC is assembled whose energy density is enhanced from 35.6 to 61.9 Wh kg^–1^ at a power density of 1.5 kW kg^–1^ under visible light (Figure 11). Besides, the authors demonstrated that the photogenerated electrons were transferred from NiO to FeCo_2_O_4_ in the direction of the built-in electric field and then injected into the external circuit. Meanwhile, the photogenerated holes participate in the charging process.

**Figure 12:**
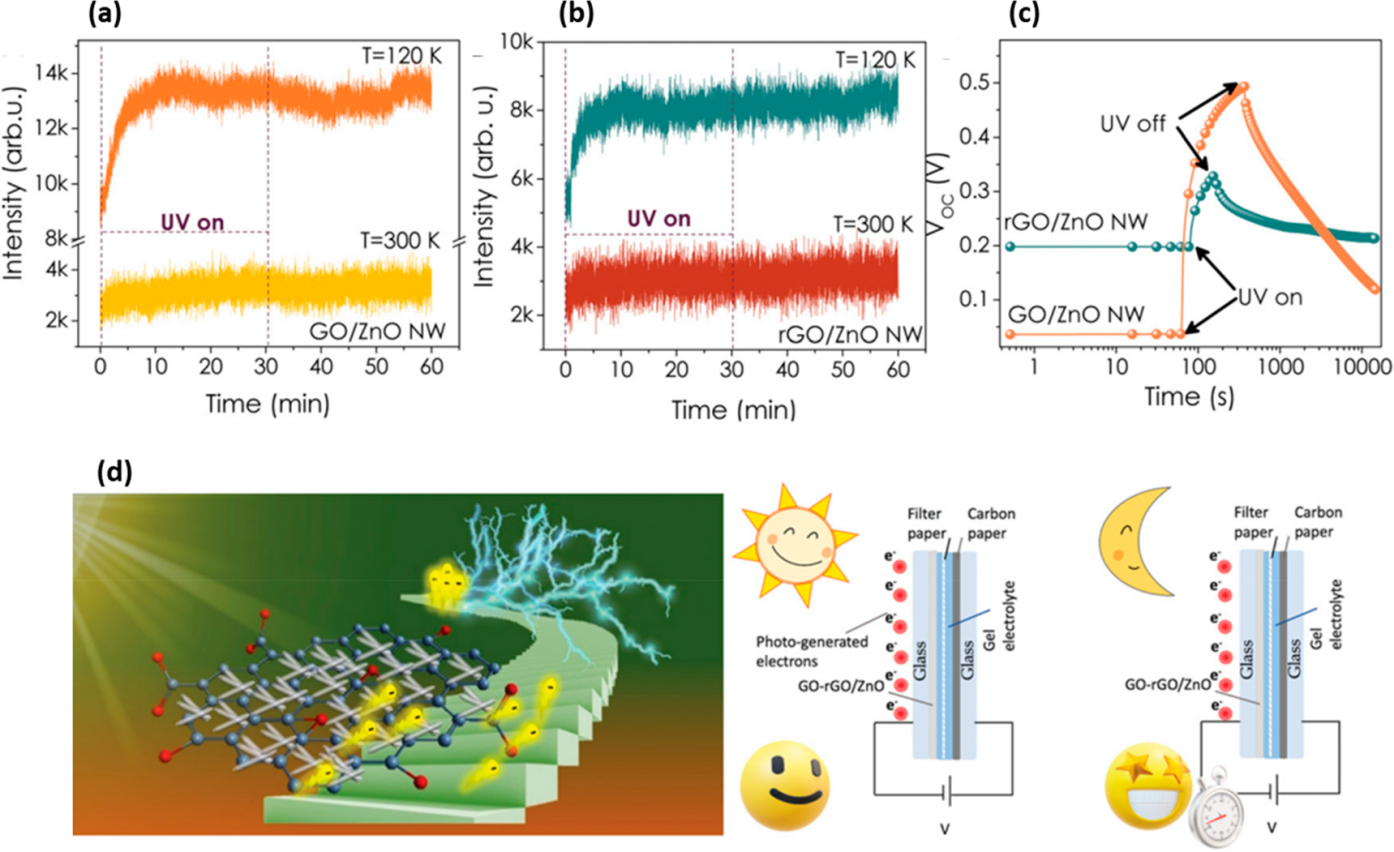
EPR data of (a) GO/ZnO NW and (b) rGO/ZnO NW with UV on–off condition at 120 and 300 K temperatures, (c) the change in *V*_OC_ of PSC devices upon UV on–off condition, and (d) schematic illustration of the photoactive material having exceptional charge trapping ability. Reproduced with permission from ref (151). Copyright 2022 Royal Society of Chemistry.

Recently, composite materials based on defective ZnO and carbon structures were explored for their potential applications in the PSC field.^54,151,168^ These composites own good light absorption properties so that they form light-generated electron–hole pairs, which increase the charge amount stored on the electrodes. Also, defect states lower the fast recombination of electrons and holes, which increases charge transport and accelerates the activity.^25,169^ The photoelectrochemical properties of these composite materials benefit from the specific defects of ZnO (vacancy and interstitial), carbonaceous materials, and 2D defects. Altaf et al.^54^ showed the connection between ZnO–GO composite defect state concentration, photogenerated defect’s reaction constants, and conversion–storage properties. In this study, electron paramagnetic resonance spectroscopy (EPR) was used as an enhanced method for defect state investigation both in the dark and under light irradiation. The EPR technique evidenced a charge-trapping phenomenon in the ZnO–GO composite. The photogenerated electrons from ZnO are easily captured by GO and stored in the π–π network, thereby increasing the ZnO capacitive behavior. Consequently, high performance and good GO/ZnO nanowire (NW)-based PSC stability were observed (Figure 12).

## 5 Current Challenges and Future Applications

The energy utilized from PVs is considered one of the most sustainable energies because of the high power of solar radiation. PV systems consisting of storage units to store electrical energy in the form of chemical energy are powerful tools to cope with the intermittent nature of solar radiation. Batteries are low-cost and abundant materials to build conventional solar energy systems. Compared with supercapacitors, the batteries present high discharging efficiency, higher energy storage density, and slower charge–discharge cycles because the electrochemical reaction takes longer to release electrons within the batteries. However, supercapacitors have low internal resistance and faster charge–discharge rates, and thus, higher cycling stability. Because of their rapid charging, supercapacitors perform better than a conventional battery. For instance, it was reported that a Li-ion battery with a 20% initial state of charge (SOC) took 12.1708 h to become a full charge, while a supercapacitor took 6.4679 s under constant solar insolation (1000 W/m^2^).^170^

The importance of dual-acting electrodes is indisputable regarding ease of use in photosupercapacitors and integration into autonomous systems. However, there is a strong need to increase the energy density of these all-in-one devices to achieve the requirements of real operating systems. Therefore, like supercapacitors, the specific capacitance of these systems must be improved. This increase can be attributed to fundamental factors, such as the surface area of the electrode materials, pore diameter, the presence of functional groups, and the improvement of their electrical properties, thereby leaving aside the light absorption properties. Also, the operating voltage should be increased to achieve the energy requirement. Although this is related to the properties of the electrolyte solution, it is also related to the design of the electrodes. In other words, working with asymmetric or battery-type hybrid designs increases the operating voltage. The optical properties of the electrodes also need to be improved to increase the performance of photosupercapacitors. Metal oxides have been used in many studies reported to date. However, these materials absorb light in the UV region because of their wide band gap, and their electrical conductivity may be low. Therefore, it is necessary to study the heterojunctions of metal oxide structures with other semiconductor materials in a way that can absorb in the visible and even infrared regions.

There are a limited number of recent studies on hybrid or battery-type electrochemical energy storage devices that can be assisted by solar energy. However, it is understood from these studies that the operating voltages and, thus, the energy density of photosupercapacitors can be increased with these configurations. Therefore, by improving other parameters of such devices, such as stability and specific capacitance, and making the device structures more suitable for practical applications, it may be possible for devices that convert and store solar energy to enter our daily lives. Another important feature of the electrode materials to be developed for these purposes is that they consist of elements abundant in nature, which is an important factor in the cost axis of mass production. Furthermore, considering that almost all of these materials contain defects in their structures, it can be thought that more experimental and theoretical studies will be carried out in the coming years on the place and importance of these defect structures in photosupercapacitor applications where optical and electrical, electrochemical, and surface interactions are at the forefront. Thanks to the progress achieved by meeting all these requirements, it is without doubt that in the coming years more autonomous devices working off-grid that meet their power needs with photosupercapacitors in many different areas, such as wearable electronics, robotic applications, and hydrogen production, will be seen.

## 6 Conclusions

This study summarizes the comprehensive, cutting-edge technologies developed in recent years on photosupercapacitors, the newest energy conversion and storage device family members. Although photosupercapacitors have been studied for about 20 years, when we look at the studies carried out in the last five years, it is seen that the diversity of materials and designs has increased. Besides, the efficiency has increased, and new promising generation devices for practical applications have been produced. It can be expected that more research will be conducted on the commercialization potential in the coming years, and scientific research and development will be shaped in this direction.

Apart from the systems in which solar cells are produced separately and integrated into supercapacitors, compact three- and two-electrode systems are also summarized in this study. We aim to draw attention to this field with successful examples of dual-effect all-in-one devices and solar-assisted battery-type or hybrid supercapacitors. It also should be realized that the optical, electrochemical, surface, and electrical properties of electrode materials, as well as their defect structures, affect their performance. In other words, the photosupercapacitor’s performance can be enhanced by inducing various types of defects (point or planar defects) in the host semiconductor. Point defects narrow the band gap, which extends the optical response to the visible range and assures a better separation of the photogenerated electron–hole pairs. Intrinsic and extrinsic defects decrease the activation energy of electrochemically active species, thereby inducing localized electrons that act as functional sites capable of adsorbing guest ions to enhance the host materials’ electrical properties. Moreover, the extrinsic defects provide multiple redox reactions that enhance the electrolyte diffusion into the crystal lattice, which increases the electrocapacitance properties. Similar to the point defects, the planar defect states lower the fast recombination of electrons and holes, which can promote electrochemical storage.
